# Non-Invasive Imaging Provides Spatiotemporal Information on Disease Progression and Response to Therapy in a Murine Model of Multiple Myeloma

**DOI:** 10.1371/journal.pone.0052398

**Published:** 2012-12-26

**Authors:** Simone S. Riedel, Anja Mottok, Christian Brede, Carina A. Bäuerlein, Ana-Laura Jordán Garrote, Miriam Ritz, Katharina Mattenheimer, Andreas Rosenwald, Hermann Einsele, Bjarne Bogen, Andreas Beilhack

**Affiliations:** 1 Department of Medicine II, Würzburg University Clinics, Würzburg, Germany; 2 Graduate School of Life Sciences, GK Immunomodulation, Würzburg, Germany; 3 Institute of Pathology, Würzburg University, Würzburg, Germany; 4 Centre for Immune Regulation, Institute of Immunology, University of Oslo and Rikshospitalet Oslo University Hospital, Oslo, Norway; 5 Interdisciplinary Center for Clinical Research (IZKF), Würzburg University, Würzburg, Germany; University of Campinas, Brazil

## Abstract

**Background:**

Multiple myeloma (MM) is a B-cell malignancy, where malignant plasma cells clonally expand in the bone marrow of older people, causing significant morbidity and mortality. Typical clinical symptoms include increased serum calcium levels, renal insufficiency, anemia, and bone lesions. With standard therapies, MM remains incurable; therefore, the development of new drugs or immune cell-based therapies is desirable. To advance the goal of finding a more effective treatment for MM, we aimed to develop a reliable preclinical MM mouse model applying sensitive and reproducible methods for monitoring of tumor growth and metastasis in response to therapy.

**Material and Methods:**

A mouse model was created by intravenously injecting bone marrow-homing mouse myeloma cells (MOPC-315.BM) that expressed luciferase into BALB/c wild type mice. The luciferase in the myeloma cells allowed in vivo tracking before and after melphalan treatment with bioluminescence imaging (BLI). Homing of MOPC-315.BM luciferase+ myeloma cells to specific tissues was examined by flow cytometry. Idiotype-specific myeloma protein serum levels were measured by ELISA. *In vivo* measurements were validated with histopathology.

**Results:**

Strong bone marrow tropism and subsequent dissemination of MOPC-315.BM luciferase^+^ cells *in vivo* closely mimicked the human disease. *In vivo* BLI and later histopathological analysis revealed that 12 days of melphalan treatment slowed tumor progression and reduced MM dissemination compared to untreated controls. MOPC-315.BM luciferase^+^ cells expressed CXCR4 and high levels of CD44 and α4β1 *in vitro* which could explain the strong bone marrow tropism. The results showed that MOPC-315.BM cells dynamically regulated homing receptor expression and depended on interactions with surrounding cells.

**Conclusions:**

This study described a novel MM mouse model that facilitated convenient, reliable, and sensitive tracking of myeloma cells with whole body BLI in living animals. This model is highly suitable for monitoring the effects of different treatment regimens.

## Introduction

Multiple myeloma (MM) is a B-cell neoplasia characterized by clonal expansion of malignant plasma cells in the bone marrow compartment. MM causes significant morbidity and mortality; it accounts for 1% of all malignant tumors. Among hematologic malignancies, MM comprises 10–15%, and it causes 20% of deaths related to cancers of the blood and bone marrow (BM) [1]. The disease mainly occurs in older people, with a median age at diagnosis of 69 years [2]. In the European Union, the MM incidence in 2008 was 16,400 for males and 15,600 for females; this corresponded to an age-standardized incidence rate of 5.4 for males and 3.9 for females per 100,000 individuals. The age-standardized mortality rate was 3.3 for males and 2.3 for females per 100,000 individuals [3].

MM and its precursor stages originate exclusively from post-germinal center B-cells, based on the observation that the immunoglobulin (Ig) sequences in MM plasma cells are somatically hypermutated and remain constant throughout the disease [4], [5]. Chromosomal abnormalities, considered a hallmark of MM, are present in nearly all patients with MM [6]. The diagnosis of MM is based on clinical symptoms, including the CRAB criteria: increased serum **c**alcium levels, **r**enal insufficiency, **a**nemia, and **b**one lesions (osteolytic lesions or osteoporosis with compression fractures). Other clinical symptoms include hyperviscosity, amyloidosis, and recurrent bacterial infections. Additionally, patients commonly exhibit more than 30% clonal bone marrow (BM) plasma cells and the presence of monoclonal protein is detected in serum and/or urine [6], [7].

Recently, survival for patients with MM has improved with the introduction of novel agents including thalidomide and its analogue lenalidomide (immunomodulatory drugs) and bortezomib (a proteasome inhibitor) [7], [8]. Nevertheless, patients in the United States diagnosed with MM between 2001 and 2007 had a 5-year relative survival rate of only 41% [9]. This clearly underlines the necessity for the development of new drugs and better treatment strategies. To that end, it would be highly desirable to develop a MM mouse model that facilitated sensitive, reliable methodologies for monitoring tumor growth and metastasis in response to different therapies.

At present, several humanized MM models are available, including the severe combined immunodeficiency SCID-hu/rab xenograft models. The SCID-hu/rab models were created by implanting either a human fetal or rabbit bone subcutaneously into an immunocompromised mouse and injecting primary human myeloma cells into the bone [10], [11]. Other MM models that use human MM cell lines also rely on immunocompromised SCID mice as recipients [12]. The drawback of these models is that it is difficult to create the xenograft mouse, and thus, it is difficult to obtain a sufficient number of mice to perform the experiments. Moreover, the use of immunocompromised mice precludes studies on the effects of potential immune system-tumor-drug interactions.

Several spontaneously occurring murine MM cell lines (5T cell lines) have also been employed; these cells can be inoculated into immunocompetent C57BL/KaLwRijHsd mice [13], [14]. Additionally, the mineral oil-induced plasmacytoma cell (MOPC-315) line has proven useful in studying immune–tumor cell interactions and drug testing in models where these murine MM cells were injected either intravenously or subcutaneously [15]–[19]. The readout of tumor burden is typically based on parameters from histology or the levels of paraprotein in the serum [14]. However, histology requires sacrificing the mice, and paraprotein levels do not provide information about tumor localization.

The present study aimed to establish an orthotopic MM mouse model injected with luciferase (luc^+^) expressing MOPC-315 cells to facilitate non-invasive, *in vivo*, tumor growth monitoring. Monitoring was performed with a light-sensitive charge-coupled device (CCD) camera system [20]–[23]. This MM model mimicked the human disease in the late stages of MM progression and allowed reliable, sensitive detection of the response to therapy.

## Materials and Methods

### Ethics Statement

All experiments were performed according to the German regulations for animal experimentation. The study was approved by the Regierung von Unterfranken as the responsible authority (Permit Number 55.2-2531.01-76/10 and -103/11).

### Mice

All experiments were performed with female BALB/c (haplotype H-2^d^) mice, 8–10 weeks old (Charles River, Sulzfeld, Germany).

### MOPC-315.BM luc^+^ Cell Line and Culture Conditions

We obtained mouse myeloma MOPC-315 cells [24], an *in vitro*-adapted cell line, from ATCC (Manassa, VA). The MOPC-315.4 cell line was derived by repeated subcutaneous (s.c.) injections of MOPC-315 cells into BALB/c mice [25]. The MOPC-315.4 cells (2×10^6^) were then injected intravenously (i.v.) into BALB/c mice. Tumor cells were flushed from the femurs of paraplegic mice, then cultured *in vitro*, and re-injected i.v. After nine *in vivo/in vitro* cycles, a cell line with bone marrow tropism, MOPC-315.BM, was obtained (B. Bogen and co-workers). MOPC-315.BM cells were co-transfected with the pGL3-Control plasmid that carried the firefly luciferase (luc) gene (Promega) and the pcDNA 3.1 plasmid that carried the neomycin resistance gene (Invitrogen). Transfected cells were cloned by limiting dilution, resulting in the MOPC-315.BM luc^+^ cell line [26].

MOPC-315.BM luc^+^ cells were cultured in RPMI, supplemented with 10% FCS, 1% penicillin and streptomycin, and 1% L-glutamine (Invitrogen, Darmstadt, Germany). Cells were maintained in a humidified incubator at 37°C and 5% CO_2_.

### Drug Preparation

Melphalan was dissolved in 100% ethanol to a concentration of 20 mM. Based on the individual weight of each mouse, the appropriate amount of melphalan was diluted in 100 µl phosphate buffered saline (PBS) to achieve a final melphalan concentration of 5 mg/kg body weight for each intraperitoneal (i.p.) injection [27], [28]. Vehicle-injected control animals received corresponding amounts of ethanol; untreated mice were not injected.

### Multiple Myeloma Tumor Model and Drug Treatment

MOPC-315.BM luc^+^ cells (H-2^d^ haplotype) were suspended (10^5^ cells) in PBS and injected i.v. via the lateral tail vein into BALB/c (H-2^d^) mice. 19 days after the injection of the MM cell line MOPC-315.BM luc^+^, animals were imaged with *in vivo* bioluminescence (BLI). After BLI, the mice were treated with melphalan (day 0 of treatment). Mice also received melphalan on days 3, 7, and 11 of treatment. The BLI measurement on day 0 represented untreated animals with the initial tumor burden before drug intervention. BLI signals measured on day 0 of treatment were set to 1, and all subsequent BLI measurements were expressed as the fold change relative to this initial signal.

### Bioluminescence Imaging (BLI)

BLI was performed on mice with an IVIS Spectrum (Caliper-Xenogen, Alameda, CA, USA) as previously described [21]. Briefly, mice were anesthetized i.p. with a mixture of ketamine (100 mg/kg) and xylazine (10 mg/kg) in PBS. Luciferin (150 mg/kg) was coinjected, and BLI measurements were started exactly 10 min later. To confirm localization of light emitting foci, at the end of the experiment mice were then euthanized, organs were prepared, and *ex vivo* imaging was started exactly 10 min after the injection. Imaging data was analyzed with Living Image 4.0 (Caliper-Xenogen, Alameda, CA, USA) and Prism 5 software (GraphPad, La Jolla, CA, USA).

Untreated or vehicle-treated mice were analyzed on days 11, 19, and 33 after the MOPC-315.BM luc^+^ cell injection to determine the average number of skeletal foci per mouse and the percentage of mice that displayed signals in liver and spleen. Individual skeletal spots and signals from the spleen and liver were visually determined on BLI images acquired from ventral and dorsal views.

### Organ Preparation and Flow Cytometry Analysis

At 42 days after the i.v. injection of 1×10^5^ MOPC-315.BM luc^+^ cells, mice were euthanized, and spleen and femur/tibia BM cells were harvested for flow cytometry analysis. Spleens were passed through a 70 µm cell strainer (BD Biosciences, Bedford, MA, USA) to create a single cell suspension, and erythrocytes were lysed. Femur and tibia BM were flushed by ejecting PBS from a syringe. Cells were stained with the following fluorophore-conjugated antibodies (clones): anti-CD4 APC-Cy7 (GK1.5); anti-CCR7 APC (4B12); anti-CD62L APC-Cy7 (MEL-14); anti-α4β7 PE (DATK32); anti-α4 (CD49d) Alexa647 (RI-2); anti-CXCR3 APC (CXCR3-173); anti-CD44 PE (IM7); anti-CD4 PE (RM4-5) (all from Biolegend); anti-CCR2 APC (475301) (R&D Systems); anti-CXCR4 PE (2B11) or anti-CXCR4 APC (2B11) (from eBioscience), and anti-CD138 Biotin (281-2) (from BD Pharmingen). Biotinylated antibodies were detected with streptavidin eFluor 450 (from eBioscience) or Alexa 488 (from Invitrogen). Dead cells were excluded with propidium iodide staining.

Flow cytometry was performed on a FACS Canto II (BD, Heidelberg, Germany), and data was analyzed with FlowJo (Tree Star, Ashland, OR, USA) and Prism 5 software (GraphPad, La Jolla, CA, USA). MOPC-315.BM luc^+^ cells were identified by first gating on CD138^+^CD4^+^ double-positive cells; both markers were constitutively expressed in both MOPC-315 and MOPC-315.BM luc^+^ cells. Within this gate, the mean fluorescence intensity (MFI) from antibodies bound to homing receptors was determined relative to the fluorescence minus one (FMO) sample (unstained cells). The FMO was used to determine the percentage of cells that expressed the homing receptors or the fold change in expression [29]. MFI were also determined for cell surface markers that displayed gradual changes in expression, including CXCR4, CXCR3, CD44, CCR2, and CCR7.

To identify α4β1^+^ and α4β7^+^ MOPC-315.BM luc^+^ cells, gates for the homing receptors, α4β7 and α4, were set based on the FMO method (**Figure S1**) [29]. The simultaneous staining of α4β7 and α4 allowed identification of α4β1^+^ cells [30]. The α4 subunit must associate with either the β1 or the β7 integrin subunit to form a functional receptor; therefore, cells that stained for α4, but not α4β7, were considered α4β1 positive.

### Fluorescence-activated Cell Sorting (FACS)

To select MOPC-315.BM luc^+^ cells that expressed either low or high levels of CXCR4, cells were stained with anti-CXCR4 APC (2B11; eBioscience). Cells were sorted on a FACS Aria III (BD, Heidelberg, Germany) and the 10% with the lowest (CXCR4^low^) and 10% with the highest (CXCR4^high^) signals were isolated. Subsequently, 5×10^5^ CXCR4^high^ or CXCR4^low^ MOPC-315.BM luc^+^ cells were injected i.v. into female BALB/c mice. After 10 days, mice were imaged with BLI, sacrificed, and spleen and femur/tibia marrow cells were extracted for FACS analysis to determine CXCR4^high^ and CXCR4^low^ expression. Additionally, cells from each sorted fraction of CXCR4^low^ and CXCR4^high^ cells were maintained in culture for 2 days, and then reanalyzed for CXCR4 expression with the same anti-CXCR4 antibody that was used for cell sorting.

### Histology

For histological examination, samples were fixed in 4% neutral-buffered formalin or embedded in Tissue-Tek OCT compound and frozen for storage. Subsequently, bones were decalcified for 72 h in formic acid (Merck, Darmstadt, Germany) and then embedded in paraffin. Samples were cut into sections (2 µm), stained with hematoxylin and eosin (H&E), and reviewed by an experienced pathologist (A.M.). In spleen and BM biopsies the total percentage of tumor infiltration was determined. In liver and lung samples, the average number of tumor cells was determined. In both cases, cells were counted under a microscope, in ten high power fields (HPF, magnification ×400). Representative images were obtained with a DP26 camera (Olympus, Hamburg, Germany) and cellSens Entry 1.5 Software, version XV 3.5.

Frozen sections were fixed in acetone and subsequently stained with anti-CD31 Alexa647 (390, Biolegend), anti-CD4 Alexa488 (RM4-5, Biolegend), and anti-IgA Biotin (11-44-2, eBioscience), followed by Streptavidin Alexa546 (Invitrogen). Nuclei were stained with 4′,6-diamidino-2-phenylindole (DAPI). Images were obtained with an AxioCamMR3 camera, mounted on an Axio Imager.Z1 microscope (Carl Zeiss, Jena, Germany) equipped with AxioVision software. The objective was a 40×/1.30 oil EC Plan-Neofluar.

### ELISA

Mouse blood samples were obtained from tail veins. After clotting, the blood was centrifuged, and the serum was removed and stored at −80°C until further use. The MOPC-315 idiotype-specific, secreted IgA M315 protein [25] was detected by ELISA. Briefly, 96 well ELISA plates were coated with 2 µg/ml idiotype-specific antibody (clone AB2.1.-4) in PBS, and plates were incubated overnight at 4°C. Duplicate serum samples were diluted 1∶100 in ELISA buffer. An M315 protein standard was prepared at 400 ng/ml and several 2-fold serial dilutions (ranging from 400 to 0.39 ng/ml). Samples and standards were added to the plates and incubated for 2 h at 37°C. Three washes with ELISA wash buffer (Biolegend) were performed. Biotinylated, rat, anti-mouse IgA (1 µg/ml; clone C10-1, BD Pharmingen) was added for 1 h at 37°C. Three washes with ELISA wash buffer were performed. Then, streptavidin-conjugated alkaline phosphatase (GE Healthcare), diluted 1∶3000 in ELISA buffer, was added, and the incubation continued for 1 h. Three washes with ELISA wash buffer were performed. Next, phosphatase substrate (Sigma-Aldrich) was added at 1 mg/ml in substrate buffer (97 ml diethanolamine, 800 ml distilled water, 101 mg MgCl_2_×6H_2_O, 200 mg sodium azide, add 10 ml 37% HCl and fill up with distilled water to 1 l). After a 25-min incubation at room temperature, absorbance was measured at 405 nm. The data was analyzed with Prism 5 software (GraphPad, La Jolla, CA, USA). Serum was collected from melphalan-treated and vehicle control mice after 14 days of treatment to compare IgA M315 serum levels to the whole body BLI signal (ventral signal+dorsal signal). For long-term observations, untreated mice were regularly bled for serum collections and simultaneously underwent BLI from ventral and dorsal views.

### Statistics

Statistical analysis was performed with InStat 3.00 or Prism 5 software (GraphPad, La Jolla, CA, USA). To determine tumor growth over time, at each timepoint, BLI data were compared among the three mouse groups with a two way ANOVA and Tukey post test for multiple comparisons. Adjusted p values are stated.

The Kruskal-Wallis test and Dunn post test were applied for comparisons among the three groups. A two tailed t-test was used to analyze the statistical significance of differences between two groups. All measurements are expressed as the mean with standard deviation (SD), unless stated otherwise.

## Results

### Effective Migration of MOPC-315.BM luc^+^ Myeloma Cells to Hematopoietic Compartments

**Figure 1:**
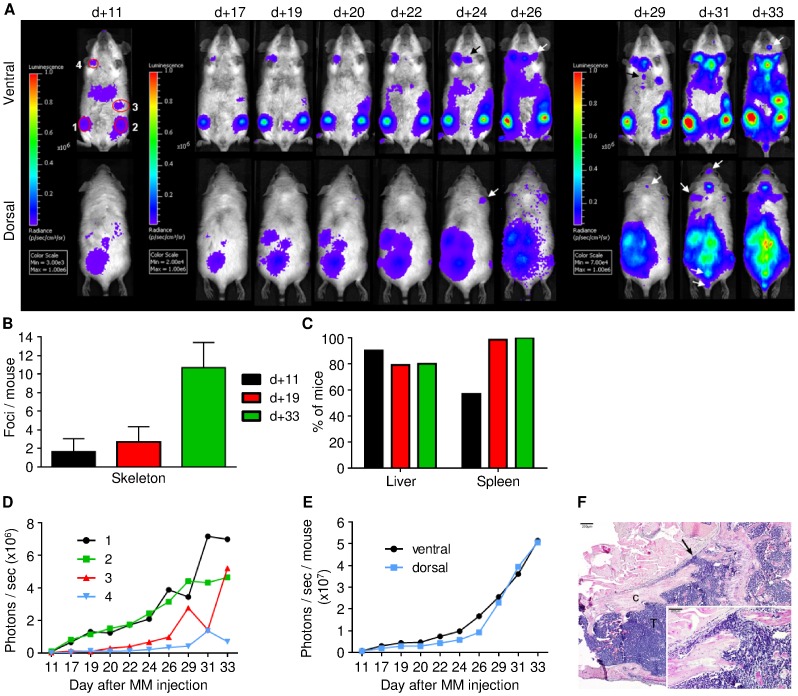
Engraftment and growth dynamics of MOPC-315.BM luc^+^ myeloma cells in vivo. BALB/c wild type mice were injected with 1×10^5^ MOPC-315.BM luc^+^ cells via the tail vein. Tumor growth and spread was regularly monitored by BLI. (**A**) BLI images of one representative mouse at indicated time points after MM injection from ventral (top) and dorsal (bottom) view. Additional emerging tumor foci over time are marked with black or white arrows. (**B**) Number of skeletal spots per mouse on days +11 (n = 51), 19 (n = 56) and 33 (n = 25) and (**C**) percentage of mice presenting signals from liver and spleen. (**D**) Quantification of single tumor foci over time as marked in (A): 1 and 2 = BM compartment of femur/tibia, 3 = spleen, 4 = BM compartment of shoulder. (**E**) Absolute signal quantification by whole body BLI from ventral and dorsal views. (**F**) Representative osteolytic lesion in the neck of femur 42 days after MM injection. Corticalis is marked as c which is destroyed (arrow) by MOPC-315.BM luc^+^ cells marked with T. Original magnification 40X, scale bar is 200 µm. Insert: original magnification 200X, scale bar is 100 µm.

To investigate the behavior and biology of MM cells in the context of the living organism luciferase expressing MOPC-315.BM luc^+^ cells were injected (i.v.) into BALB/c recipients. First, MM cell homing patterns, expansion, and dissemination were tracked with sequential, non-invasive, BLI. At 11 days after the injection, the MOPC-315.BM luc^+^ cells in all recipients emitted discrete signals (**Figure 1A**). Light-emitting foci appeared most frequently in the skull, vertebrae, sternum, femur, tibia, and other parts of the skeleton. Mice exhibited an average of 1.6 skeletal bioluminescent foci on day +11 (**Figure 1B**), and a signal was detected in the spleen in 57% of mice (**Figure 1C**) (n = 51, 3 independent experiments). Therefore, early homing sites of MOPC-315.BM luc^+^ cells primarily comprised the hematopoietic compartment.

From the initial proliferation hot spots, MM cells metastasized to other skeletal areas (**Figure 1A** **arrows**). Between days +19 (n = 56, 3 independent experiments) and +33 (n = 25, 3 independent experiments), the average number of skeletal MM foci increased from about 3 to over 10 per mouse (**Figure 1B**). After day +19, signals were detected in the spleen in nearly all mice (**Figure 1C**). Of note, signals were detected in the liver in 90% of mice on day +11; however, unlike the skeletal or splenic foci, the liver signals remained comparatively low-intensity, and they did not increase in signal strength throughout the experiment (**Figure 1A**). Four representative spots (**Figure 1A**) were marked on day 11 to determine whether the increase in tumor burden over time was due to growth of an initial focal tumor or proliferation and dissemination of multiple myelomas. The signal intensities were measured for each representative spot over time (**Figure 1D**). Spots 1 and 2 represented signals in the femur, spot 3 was in the spleen, and spot 4 was in a shoulder bone. Indeed, local signals increased at these four spots over time (**Figure 1D**). This initial, persistent, focal tumor growth was followed by subsequent spreading to distant skeletal regions, which resulted in a steady increase in the total luciferase signal (**Figure 1E**). Bone remodeling and osteolytic lesions were detected in >35% of animals at 42 days after MM injection. For example, corticalis destruction was observed in the neck of the femur (**Figure 1F**).

### MOPC-315.BM luc^+^ Cells Express Receptors for Bone Marrow Homing and Retention

**Figure 2:**
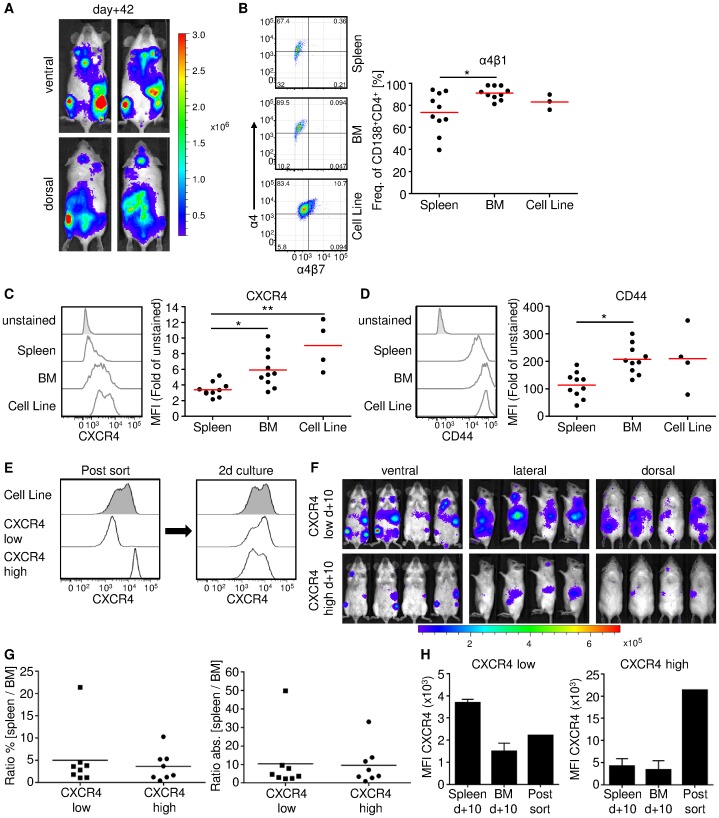
Flow cytometric measurement of surface receptors associated with BM homing and infiltration of myeloma cells. BALB/c wild type mice were injected with 1×10^5^ MOPC-315.BM luc^+^ cells via the tail vein. (**A**) 42 days after MM injection mice showed high BLI signals from hematopoietic compartments such as femur/tibia and spleen. Shown are two representative mice from ventral and dorsal view immediately before cells from BM and spleens were harvested for flow cytometry. (**B–D**) Besides BM and spleen derived MM cells, we also analysed MOPC-315.BM luc+ cells from culture. Dead cells were excluded by propidium iodide staining and MOPC cells identified as CD138^+^CD4^+^ double positive cells. (**B**) α4β1 integrin positive MOPC-315.BM luc^+^ cells were identified by flow cytometry as α4^+^ (CD49d^+^) and α4β7^−^. Representative FACS plots and the corresponding graph are shown, stating the frequency within CD138^+^CD4^+^ MOPC-315.BM luc^+^ cells expressing α4β1. For CXCR4 (**C**) and CD44 (**D**) representative histograms for each organ and cell line, including unstained fluorescence minus one (FMO) sample are displayed. Corresponding graphs state the fold difference in mean fluorescence intensity (MFI) related to the unstained FMO sample. BM and spleen: two independent experiments, n = 10, cells from cell culture: n = 4 for CXCR4 and CD44, n = 3 for α4β1. * indicates p<0.05 and ** indicates p<0.01 as determined by Kruskal-Wallis test with Dunn post test. (**E**) MOPC-315.BM luc^+^ cells were sorted for CXCR4^low^ and CXCR4^high^ expression. After 2 days in cell culture sorted cells regained the original CXCR4 expression level of the cell line. (**F**) 5×10^5^ sorted cells were i.v. injected into 4 female BALB/c mice each and BLI from ventral, lateral and dorsal was performed 10 days later. Sorted CXCR4^low^ as well as CXCR4^high^ CXCR4 cells readily homed to the BM compartment as well as to the spleen. (**G**) After BLI the mice were sacrificed, cells from left and right femur/tibia (separately) and the spleen extracted, and percentage as well as absolute numbers for CD138^+^CD4^+^ MM cells determined. From these values a ratio of spleen/BM was calculated to determine the homing capacity of the sorted populations. (**H**) Comparison of CXCR4 expression levels of sorted CXCR4^low^ and CXCR4^high^ cells immediately before injection and MM cells from BM and spleen after 10 days *in vivo* revealed a dynamic CXCR4 regulation.

The results indicated strong tropism of MOPC-315.BM luc^+^ myeloma cells towards the hematopoietic compartment. Thus, cells were investigated to identify homing receptors that previously showed efficient recruitment to BM niches. A panel of surface markers was tested on MOPC-315.BM luc^+^ cells extracted from BM and spleen at 42 days after MM injection (n = 10, two independent experiments) (**Figure 2A**). For comparison the surface markers were also tested in MOPC-315.BM luc^+^ cells maintained in from tissue culture (n = 3; 4 independent measurements).

Expression of α4β1 integrin was detected in 80% of the *in vitro* cells, in 91% of the MOPC-315.BM luc^+^ cells reisolated from the BM, and in 73% of cells reisolated from the spleen (p<0.05) (**Figure 2B**). Integrin α4β1 facilitates transendothelial migration of MM cells into the BM. Integrin α4β1 can be upregulated by the interaction between CXCR4 and its ligand, stromal cell-derived factor-1 (SDF-1) [31], [32], a chemoattractant constitutively produced by BM stromal cells. Thus, MM cells that express CXCR4 are recruited and retained in the BM [32]. Of note, MOPC-315.BM luc^+^ cells from the spleen exhibited downregulated CXCR4 expression compared to those isolated from the BM (p<0.05) and those maintained in cell culture (p<0.01) (**Figure 2C**).

The CD44 receptor mediates MM cell binding to hyaluronic acid on the BM endothelium; this interaction supports BM homing and invasion [33], [34]. Overall CD44 expression was high in MOPC-315.BM luc^+^ cells, both *in vitro* and in different compartments *in vivo*. Nevertheless, MOPC-315.BM luc^+^ cells isolated from the BM expressed significantly higher CD44 levels than those in the spleen (p<0.01) (**Figure 2D**).

CXCR4^low^ and CXCR4^high^ expressing MOPC-315.BM luc^+^ cells were tested for preferential homing to the spleen or the BM, which might explain the result shown in **Figure 2C**. We injected 5×10^5^ of either CXCR4^low^ or CXCR4^high^ MOPC-315.BM luc^+^ cells into syngeneic mouse recipients (n = 4 each). Interestingly, when these two distinct populations of CXCR4 expressing MOPC-315.BM luc^+^ cells were maintained for 2 days in cell culture, both populations regained the initial CXCR4 expression profile (**Figure 2E**). Consistent with that finding, after 10 days *in vivo*, non-invasive BLI (**Figure 2F**) showed that signals for both sorted populations were detected in both the spleen and the BM compartment. Thus, the CXCR4 expression level of i.v. injected cells did not govern homing to the spleen or BM. However, *in vivo* BLI showed that the CXCR4^low^ MOPC-315.BM luc^+^ cells proliferated more than the CXCR4^high^ cells. After BLI, animals were euthanized, and cells from the spleen, left femur/tibia, and right femur/tibia were harvested separately for FACS analysis. The percentage and the absolute numbers of CD138^+^CD4^+^ were determined for each femur/tibia ( = BM) and the spleen. The ratios of spleen/BM signals showed that there was no difference in homing preference between CXCR4^low^ and CXCR4^high^ MOPC-315.BM luc^+^ cells (**Figure 2G**). This corroborated the *in vivo* BLI results. We also compared the MFIs of freshly sorted CXCR4^low^ and CXCR4^high^ MOPC-315.BM luc^+^ cells with the MFI of MM cells extracted from femur/tibia and from the spleen 10 days after injection (**Figure 2H**). This showed that CXCR4 expression dynamically changed *in vivo*.

The other homing receptors tested, for example α4β7 integrin, showed low expression (∼10% of MOPC-315.BM luc^+^ cells) *in vitro* and levels decreased *in vivo* (**Figure S2A**). However, because MOPC-315.BM luc^+^ cells exhibited strong homing to the spleen, we tested the expression of CD62L and CCR7 receptors, which are involved in homing to secondary lymphoid organs. CD62L expression was expressed at low levels on MOPC-315.BM luc^+^ cells prior to injection; after injection, expression increased in cells from the spleen, but not in cells from the BM (**Figure S2B**). CCR7 was not expressed at significant levels, either before or after injection (**Figure S2C**). Finally, CXCR3 and chemokine receptor 2 (CCR2), important for MM progression, dissemination [35], and BM homing [36] were not significantly expressed on the cells analyzed (**Figure S2D and S2E**).

### Attenuation of Myeloma Disease Progression in Mice Treated with Melphalan

**Figure 3:**
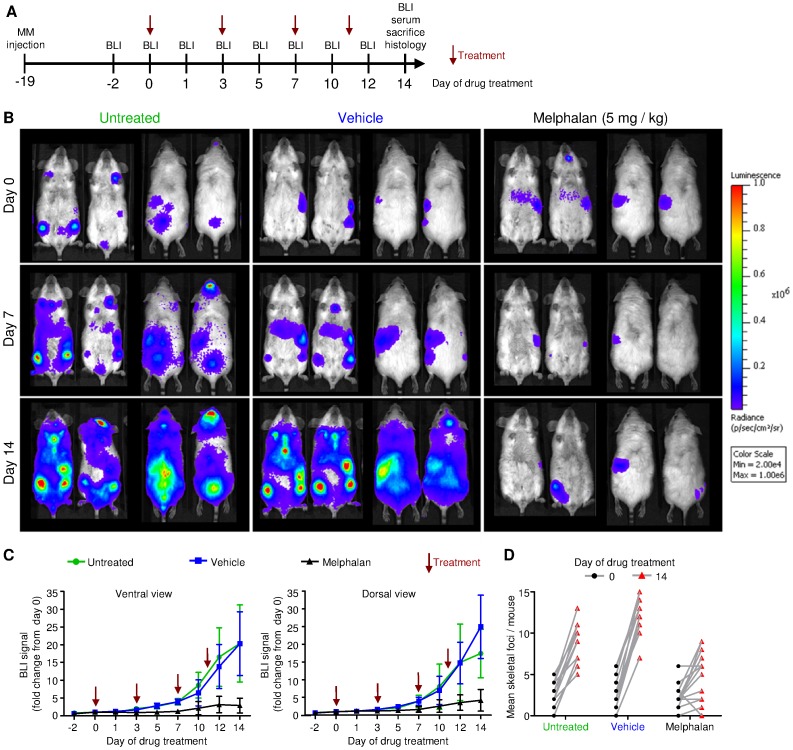
Detection of residing MOPC-315.BM luc^+^ cells and *in vivo* monitoring of reduced myeloma progression due to melphalan treatment. BALB/c wild type mice were injected with 1×10^5^ MOPC-315.BM luc^+^ cells via the tail vein. On day +19 after inoculation, tumors were established in all mice and readily detected by BLI. Then treatment was started ( = day 0 of treatment). Mice received 5 mg/kg melphalan (n = 9, two independent experiments) or mock treatment (vehicle control, n = 13, three independent experiments) intraperitoneally. One control group of MOPC-315.BM luc^+^ tumor bearing mice did not receive any treatment (untreated control, n = 14, three independent experiments). (**A**) Schematic study design, indicating treatment time points in respect to time after MM injection and end of experiment. Red arrows pinpoint melphalan treatment. (**B**) BLI images of two representative mice per group at selected time points in ventral (left) and dorsal (right) view. (**C**) Quantification of bioluminescence signal intensities over time from ventral or dorsal. Signals at day +19 were set as starting point and the following measurements were calculated as fold change of this initial signal intensity. Mice were treated at time points as indicated by arrows. Significant difference between melphalan treated mice vs vehicle control or vs untreated animals starting on day 10 of treatment for both, ventral (untreated vs melphalan p<0.0001, vehicle vs melphalan p = 0.0032) and dorsal (untreated vs melphalan p = 0.0006, vehicle vs melphalan p = 0.0024). (**D**) Quantification of skeletal tumor foci in untreated, vehicle control and melphalan treated mice on day 0 and 14 of drug treatment.

To test this mouse model for reliable detection of changes in tumor load with *in vivo* imaging, we treated tumor-bearing mice with the alkylating agent, melphalan, a well-established treatment for MM in humans. We included mice treated with melphalan (dissolved in ethanol; n = 9), a vehicle-treated control group (ethanol only; n = 13), and untreated controls (n = 14). The treatment began 19 days after the MOPC-315.BM luc^+^ cell injection, when the disease was fully established (day 0 of treatment). Mice were treated with melphalan on days 0, 3, 7, and 11 of treatment, and sacrificed for histology on day 14 of treatment (**Figure 3A**). Images (**Figure 3B**) show ventral (left) and dorsal (right) views of two mice representative of each group; BLI was performed immediately before the first treatment (day 0 of treatment), on day 7 of treatment, and immediately before sacrificing the animals (day 14 of treatment). BLI signals markedly increased in vehicle controls and untreated animals, but no significant increases were noted in mice treated with melphalan (**Figure 3C**). Starting on day 10 of treatment, significant differences between groups were detected in both ventral (untreated vs. melphalan p<0.0001, vehicle vs. melphalan p = 0.0032) and dorsal (untreated vs. melphalan p = 0.0006, vehicle vs. melphalan p = 0.0024) images. No significant differences were detected between vehicle-treated and untreated mice.

Additionally, we detected new skeletal tumor foci from disseminating MM cells in untreated and vehicle-treated mice; in contrast, melphalan-treated mice showed little or no MM dissemination (**Figure 3B**). Indeed, the numbers of visible skeletal tumor foci had considerably increased from day 0 to day 14 of treatment in both control groups. In contrast, melphalan-treated mice showed few or no new disseminations, and some exhibited a reduced number of tumor foci (**Figure 3D**).

Two mice in the untreated control group developed paralysis in the hind legs at 30 and 32 days after injection of MOPC-315.BM luc^+^ cells. This was due to tumor growth in the spine, which caused nerve compression.

*Ex vivo* BLI was performed to confirm the *in vivo* BLI signal localizations assigned to organs or bones. Mice were sacrificed on day +14 of treatment for *ex vivo* imaging. Organs that displayed *in vivo* signals (liver, spleen, and femur/tibia) were separated from organs that did not show *in vivo* signals (lungs). The signals detected with *ex vivo* BLI were consistent with those detected *in vivo* in liver, spleen, and femur/tibia (**Figure S3**). In contrast, *ex vivo* imaging revealed a signal in the lungs that was absent on *in vivo* BLIs (**Figure S3**). Similarly, *ex vivo* imaging supported the findings from *in vivo* BLI for melphalan-treated mice. Lower signal intensities were found with treatment than without treatment.

### Histological Examinations Confirmed *in vivo* BLI Measurements of Response to Drug Treatment

**Figure 4:**
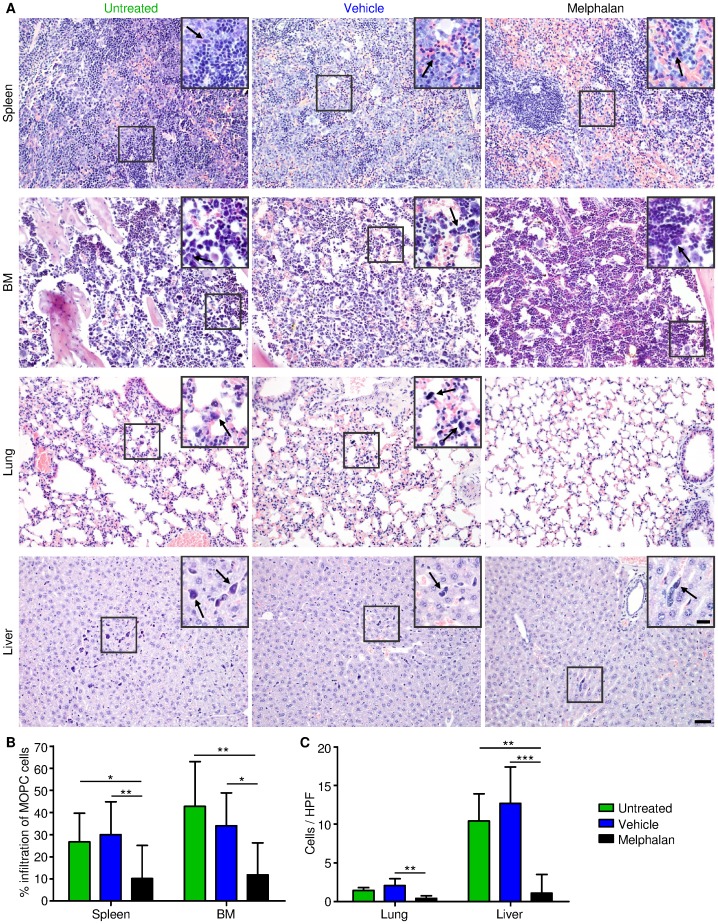
Histopathological analysis of organs from melphalan, vehicle control treated and untreated mice confirms *in vivo* BLI data. (**A**) Representative H&E stainings from organs harvested at day +33 after tumor inoculation from the different treatment groups. The hematopoietic compartments BM and spleen displayed clear infiltration of MOPC-315.BM luc^+^ cells, while MOPC-315.BM luc^+^ cells in lung and liver resided within the blood vessels as indicated by arrows. Scale bar is 50 µm for all shown sections (original magnification 200X/0.70 NA) and 20 µm for inserts (original magnification 400X/0.85 NA). (**B**) Determination of the percentage of MOPC-315.BM luc^+^ cells within BM and spleen and (**C**) number of cells per high power field (HPF) in lung and liver significantly correlates with the melphalan treatment and thereby confirms *in vivo* and *ex vivo* imaging data.

To resolve MOPC-315.BM luc^+^ cells within organs and to confirm measured BLI signals on a cellular basis, histological sections were evaluated by an experienced, unbiased pathologist. MOPC-315 luc^+^ cells were morphologically distinct from other cell types with H&E stain; therefore no additional stain was necessary (**Figure 4A**). MOPC-315.BM luc^+^ cells were observed in the spleen and femur/tibia BM. In contrast, in liver and lungs, myeloma cells were localized in blood vessels, and did not invade tissues (**Figure 4A** **, magnified inserts and Figure S4**). This suggested that the weak *in vivo* BLI signals from the vascularized liver originated from circulating MM cells that passed through the organ during the signal acquisition.

To confirm the significant differences among the three treatment groups, single MM cells were counted in liver and lung; in the spleen and BM, the percentage of MM cell infiltration was evaluated, because the large numbers of MM cells made counting unreliable. Compared to untreated controls, melphalan-treated mice clearly exhibited less infiltration in spleen, in BM (**Figure 4B**), and in the vasculatures of liver and lung (**Figure 4C**). This reduced circulation of MM cells in melphalan-treated mice might explain the reduced dissemination detected with *in vivo* BLI (**Figure 3D**).

The histological examination of the liver did not reveal any progressive disease in melphalan-treated mice. However, in the vehicle-treated group, liver infiltration was observed; in 2 out of 10 mice, very small tumors were found (less than 1 mm in diameter). In the untreated group, a liver tumor of 15 mm in diameter was observed in 1 of 9 animals. In quantifications of liver infiltration, histological liver data from the animals with liver tumors were excluded (**Figure 4C**). Most likely, the melphalan treatment inhibited the formation of tumors in the liver.

### Serum M315 IgA Levels also Verified Treatment Response Measured with Non-invasive BLI

**Figure 5:**
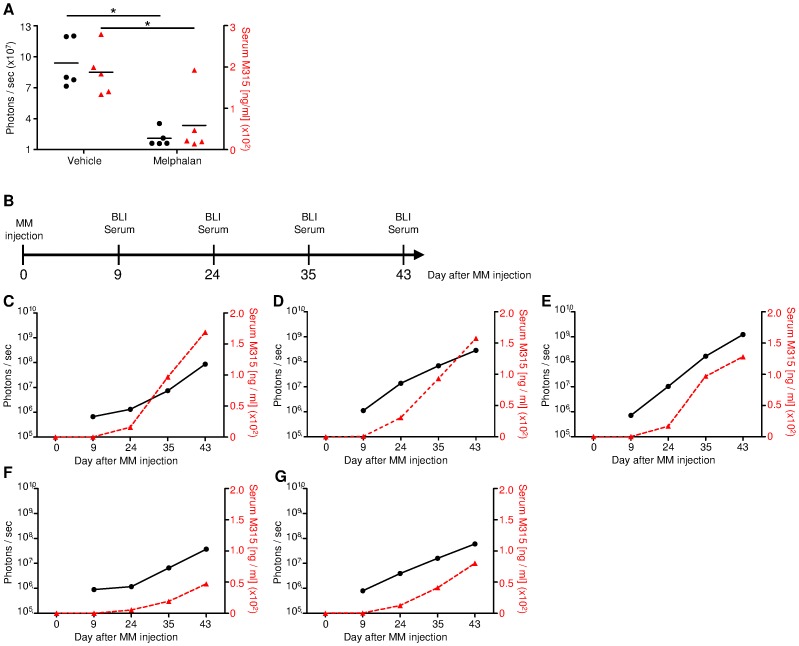
Tumor detection by *in vivo* BLI correlates to M315 IgA serum measurement by ELISA. (**A**) M315 serum levels of melphalan treated (n = 5) and vehicle control (n = 5) on day 14 of treatment and simultaneous BLI measurement of the same mice. The treatment schedule is depicted in Figure 3A. Measurement of idiotype specific M315 IgA significantly differed between the groups (two-tailed p value 0.0171) as it did with BLI (ventral+dorsal signal) (two-tailed p value 0.0221). (**B**) Scheme indicating time points for BLI measurement and serum collection for data presented in (C–G). (**C–G**) Idiotype specific M315 IgA serum levels of 5 untreated mice constantly increased over time correlating with progressing tumor burden as measured with BLI (ventral+dorsal signal). Of note, BLI measurements provided signals in early disease stages starting from day +9, whereas M315 IgA levels were not detectable at this time point. The left y-axis displays BLI signal intensity (black circles, solid lines); the right y-axis displays serum M315 IgA (red triangles, dashed lines).

The non-invasive BLI data was also verified by ELISA measurements of M315 IgA immunoglobulin secreted by MOPC-315.BM luc^+^ cells [37]. The measurement of MM-derived Ig is widely used in mouse models to determine tumor load [38], [39]. We tested whether MOPC-315-idiotype-specific IgA (M315 IgA) correlated with tumor load after drug treatment. Both *in vivo* BLI and histology indicated significantly less tumor burden in melphalan-treated vs. vehicle-injected control mice. On day 14 of treatment, serum was collected from representative mice from the vehicle and melphalan treatment groups, and non-invasive BLI was performed in parallel (**Figure 3A**). M315 IgA levels were significantly reduced in melphalan-treated mice (n = 5) compared to vehicle-treated control animals (n = 5) (two-tailed p-value 0.0171). This was supported by the simultaneously obtained *in vivo* BLI data, which showed a similar significant difference (two-tailed p-value 0.0221) (**Figure 5A**).

Five untreated mice were monitored over an extended time period with simultaneous serum M315 IgA and BLI measurements (**Figure 5B**). In general, the M315 IgA data correlated with the BLI data; three mice with high BLI signals (ventral+dorsal) also showed high M315 IgA levels (**Figure 5C–E**); two mice with low BLI signals also had comparatively low M315 IgA levels (**Figure 5F, G**). In all 5 animals, M315 IgA was clearly detected on day +24, and it steadily increased until the last measurement on day +43.

In mice, the half-life of IgA is 24 h [40]; however, in humans, the half-life is considerably longer, up to 4–6 days [41]. Therefore, in humans, M315 IgA ELISA measurements might slightly overestimate the actual tumor load due to accumulation. In contrast, BLI can detect the MM load in real time. Furthermore, the first BLI signals were detectable on day +9 after MM injection, when M315 IgA levels were at background levels. This indicated that BLI was more sensitive than serum M315 IgA for the detection of MM in this mouse model.

## Discussion

In this study, we created a reliable, highly reproducible, orthotopic, MM mouse model to facilitate *in vivo* preclinical drug testing. Moreover, because this model retained a complete, unmanipulated immune system, we believe that this model will be also suitable in future studies of immune cell–tumor cell interactions in various settings; for example, in studies of graft-versus-myeloma effects after allogeneic stem cell transplantation. To facilitate sensitive, non-invasive monitoring of tumor growth and localization, we transfected freshly isolated MOPC-315.BM cells with luciferase before injection into syngeneic recipients. This allowed *in vivo* BLI detection of MM cells throughout the body. The initial tumor inoculation and subsequent outgrowth revealed striking similarities to the progression and localization observed in the human disease. We detected typical multifocal growth in hematopoietic compartments involving the skeleton and spleen.

BLI offered the ability to detect new tumor foci, and to quantify the growth of single spots within the whole mouse. This important technique also allowed assessments of the impact of a therapy on the overall tumor burden and the changes in single foci. Thus, BLI can be used to determine the potential of new drugs on tumor metastases and disease involvement in different organ compartments. Due to the high sensitivity of BLI measurements, we could detect small increases in tumor burden throughout the body. This feature is important for assessing the efficacy of drug treatments or for detecting minimal residual disease.

Several studies have previously demonstrated strong correlations between non-invasive BLI data and the actual tumor load measured with traditional methods, including serum markers or histopathology [42], [43]. In this study, we also demonstrated the reliability of *in vivo* BLI in the MM mouse model. Differences in tumor growth were achieved by treating some mice with melphalan, the treatment of choice for patients with MM [44], [45]. Our results with *in vivo* BLI showed clear differences between treated and untreated groups. Melphalan reduced the tumor burden and diminished the tumor spread throughout the body. Subsequent *ex vivo* imaging and, more importantly, histological analyses validated the *in vivo* BLI data. These analyses demonstrated that this model was suitable for non-invasive measurements of tumor load and spread. Thus, this model permitted long-term tracking of MM cells with high sensitivity in an anatomical context, and obviated the need to euthanize mice.

This model also resembled human MM in the detectable secretion of IgA immunoglobulin from MOPC-315.BM luc^+^ cells. The idiotype-specific, M315 IgA protein was detectable within the total IgA in mouse serum. In addition to histology, serum immunoglobulin measurements are widely used to determine tumor load in mouse models [38], [39]. In this mouse model, the ELISA measurement of idiotype specific IgA correlated with BLI measurements of tumor burden. With both methods, significant differences in tumor load were detected between melphalan-treated and untreated mice. Moreover, the correlation between M315 IgA and BLI measurements was consistent over 43 days. However, the BLI signal was detected earlier (within 9 days after MM injection) than the change in M315 IgA above background. Additionally, BLI provided important information about tumor cell location, which cannot be measured with ELISA.

A strong correlation was also found between *in vivo* BLI and flow cytometry analyses (**Figure S5**).

The MOPC-315.BM luc^+^ cells showed similarity to the human MM disease in the strong homing to hematopoietic compartments. When, cells were tested for receptors associated with this homing pattern, we found that MOPC-315.BM luc^+^ cells expressed the MM-associated surface marker CD138. This molecule is typically expressed in human MM cells. Interestingly, the MOPC-315.BM luc^+^ cells also displayed the surface marker CD4, which is a typical marker for T cells, but not for B cells. However, some studies have reported cases where human MM cells acquired T cell-associated markers [46]. CD4 expression was also found in MOPC-315 parental cells obtained from the ATCC (**Figure S6**). Therefore, we concluded that this marker was not involved in the homing of MOPC-315.BM luc^+^ cells into hematopoietic compartments. Our results also showed that CXCR4 was expressed on the MOPC-315.BM luc^+^ cell line *in vitro* and *in vivo*. CXCR4 binds to the chemokine, SDF-1, produced by BM stromal cells, which triggers MM cell migration towards the BM [47]. *In vitro* cultured MOPC-315.BM luc^+^ cells expressed higher CXCR4 levels than the cells reisolated from BM and spleen. This might explain the strong BM tropism of the cell line upon i.v. injection. A similar finding was reported in patients with MM; circulating MM cells displayed higher CXCR4 levels than cells isolated from the BM [47]. Furthermore, previous studies on human and murine myeloma cells showed that SDF-1 binding to CXCR4 promoted transendothelial migration by upregulating the expression of several integrins, including α4β1 [48]. Indeed, our results showed that a high percentage of MOPC-315.BM luc^+^ cells expressed this important integrin *in vitro* and *in vivo*. Of note, MM cells expressed higher α4β1 levels in the BM than in the spleen. The CD44 receptor also was reportedly involved in MM cell infiltration of the BM through binding to hyaluronan on BM tissue [34], [48]. Several CD44 splice variants were strongly expressed in patients with MM; this provided a valuable marker for distinguishing healthy individuals from those with MM [49]. Our data also demonstrated high CD44 expression on MOPC-315.BM luc^+^ cells *in vitro* and *in vivo*. That result suggested that the cell line could interact with the host environment, and this interaction may regulate surface molecule expression to coordinate BM homing and retention.

Taken together, our findings of *in vivo* MM growth and spread in this MOPC-315.BM luc^+^ mouse model represented important criteria for the usefulness of this model in preclinical drug testing. Our results implied that the cell line retained plasticity, and could react to outside stimuli, including cell-cell interactions and drug administration. Furthermore, MOPC-315.BM luc^+^ cells expressed important BM homing receptors that are highly relevant in the human disease and are targeted by currently approved drugs. Moreover, interactions between MOPC-315.BM luc^+^ cells and the surrounding tissues were evidenced by findings of MM-typical osteolytic lesions and bone remodeling in this mouse model.

Our model provides an advantage over existing animal models, because it does not rely on the spontaneous onset of B-cell malignancies. In other animal models, it is difficult to predict the time of disease onset, disease progression, and the exact disease subtype [50]. In addition, our model provides the ability to introduce the tumor at the same time to a large number of experimental mice. This facilitates meaningful statistical analyses, and holds great promise for testing the efficacy of a drug.

This orthotopic MM mouse model closely mimicked the late stages of human MM disease. We demonstrated that it provided early detection of disease progression, a great advantage over existing animal models. The non-invasive detection of luc^+^ MM cells provided important information on the location of MM cells in an anatomical context. Therefore, this model represents a convenient, reproducible platform for preclinical drug testing.

## Supporting Information

Figure S1 **Representative flow cytometry gating scheme according to the fluorescence minus one method (FMO). (A)** First, live cells were identified using propidium iodide staining. Within the live cells the gate for CD138^+^CD4^+^ MM cells was set and applied to all samples within the measurement. Among those cells the quadrant gate for α4 and α4β7 was set according to the FMO method. The first FMO sample comprises all sampled antibodies except for α4 and the quadrant gate was set that α4 unstained cells appeared in the α4 negative quadrant. This gate was applied to the FMO sample where only α4β7 staining is missing. The gate was adjusted that α4β7 unstained cells appeared in the respective negative quadrant. This FMO gate was applied to all further samples within the measurement. **(B)** Representative samples from BM, spleen and the cell line with applied FMO gates.
(TIF)

Figure S2 **Flow cytometric measurement of surface receptors associated with BM homing and infiltration of myeloma cells.** MOPC-315.BM luc^+^ myeloma cells were either directly taken from cell culture or extracted from BM and spleen as indicated and identified as CD138^+^CD4^+^ double positive cells. α4β7 integrin positive MOPC-315.BM luc^+^ cells were identified by flow cytometry as α4^+^ (CD49d^+^) and α4β7^+^ double positive. Representative quadrant gates or histograms for each organ and cell line, including unstained fluorescence minus one (FMO) sample are shown. Graphs state the frequency within CD138^+^CD4^+^ MOPC-315.BM luc^+^ cells expressing α4β7 **(A)** or fold difference of mean fluorescence intensity (MFI) values of CD62L **(B)**, CCR7 **(C)**, CXCR3 **(D)** or CCR2 **(E)** in relation to the unstained FMO sample.
(TIF)

Figure S3 **Verification of** ***in vivo*** **signal localization by** ***ex vivo*** **BLI.** BALB/c wild type mice were injected with 1×10^5^ MOPC-315.BM luc^+^ cells via the tail vein. 19 days after inoculation tumors were established in all mice and readily detected by BLI. Then treatment was started ( = day 0 of treatment). Mice received 5 mg/kg melphalan or mock treatment (vehicle control) intraperitoneally. One control group of MOPC-315.BM luc^+^ tumor bearing mice did not receive any treatment (untreated). Mice were sacrificed on day +14 of treatment and organs were prepared for *ex vivo* BLI. Organs from one representative mouse per group are shown. *In vivo* BLI localization of signals from the liver, spleen and femur/tibia are confirmed. The signal from the lungs is only detected by *ex vivo* but not by *in vivo* BLI. Organs from the melphalan treatment group displayed lower signal intensities, indicating lower tumor burden when compared with untreated or vehicle controls. Therefore, *ex vivo* BLI corroborates *in vivo* data as well as histopathological analysis of the response to melphalan therapy.
(TIF)

Figure S4 **CD31 staining verifies MOPC-315 localization inside blood vessels in the lung.** **(A)** Representative immunofluorescence staining of an IgA^+^CD4^+^ MOPC-315.BM cell within a pulmonary CD31^+^ vessel in the lung taken from untreated mice 33 days after MM injection. **(B)** Negative control staining, without anti-CD31, anti-IgA and anti-CD4. Only secondary strepavidin Alexa546 and DAPI was added. Single color channels and merge including DAPI are shown. Blue – CD31, red – IgA, green – CD4, white – DAPI (nuclei). Scale bar is 10 µm. Original magnification 400×/1.30 NA.
(TIF)

Figure S5 **In vivo BLI measurements strongly correlate with MM burden determined by flow cytometry in spleen and femur/tibia.** To additionally verify that non-invasive BLI data correlate with actual MM load we measured *in vivo* BLI signals from the spleen and femur/tibia and subsequently extracted cells from both organs for FACS analysis. For FACS MM cells were identified among living cells as CD4^+^CD138^+^. The measured percentage of MM cells infiltrating the spleen or bone marrow compartments was correlated to measured BLI signals using a Pearson correlation. Femur/tibia: Pearson r = 0.9817, p (two tailed) <0.0001; Spleen: Pearson r = 0.9757, p (two tailed) <0.0001.
(TIF)

Figure S6 **CD4 expression of MOPC-315.BM luc^+^ and the wild type (WT) cell line.** MOPC-315.BM luc^+^ and MOPC-315 WT cells (obtained from ATCC) were stained for CD4. Only live cells as determined by propidium iodide staining were used for the analysis. Both cell lines clearly expressed CD4 to the same extent. Therefore, the constitutive CD138 and CD4 co-expression can be considered as a hallmark to uniquely identify these cells. Grey tinted histogram shows unstained luc^+^ or WT cells respectively, black histogram shows CD4 stained cells.
(TIF)
