# Supplementary figures and images for: Non-Invasive Imaging Provides Spatiotemporal Information on Disease Progression and Response to Therapy in a Murine Model of Multiple Myeloma
Source: PLoS One. 2012 Dec 26;7(12):e52398. doi: 10.1371/journal.pone.0052398 (PMC3530556; doi:10.1371/journal.pone.0052398)

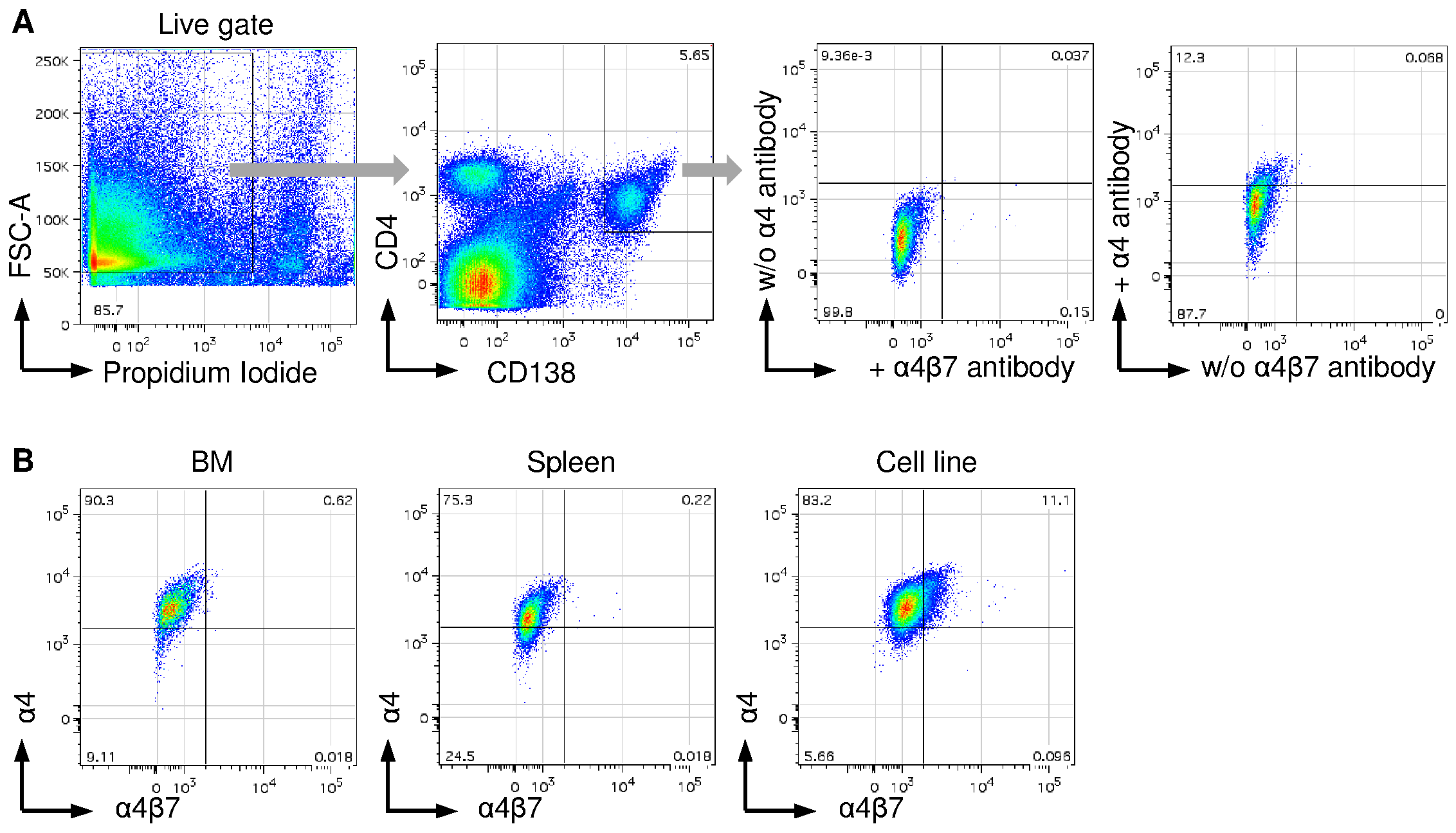

Supplement: Figure S1 — Representative flow cytometry gating scheme according to the fluorescence minus one method (FMO). (A) First, live cells were identified using propidium iodide staining. Within the live cells the gate for CD138+CD4+ MM cells was set and applied to all samples within the measurement. Among those cells the quadrant gate for α4 and α4β7 was set according to the FMO method. The first FMO sample comprises all sampled antibodies except for α4 and the quadrant gate was set that α4 unstained cells appeared in the α4 negative quadrant. This gate was applied to the FMO sample where only α4β7 staining is missing. The gate was adjusted that α4β7 unstained cells appeared in the respective negative quadrant. This FMO gate was applied to all further samples within the measurement. (B) Representative samples from BM, spleen and the cell line with applied FMO gates. (TIF) [file pone.0052398.s001.tif]

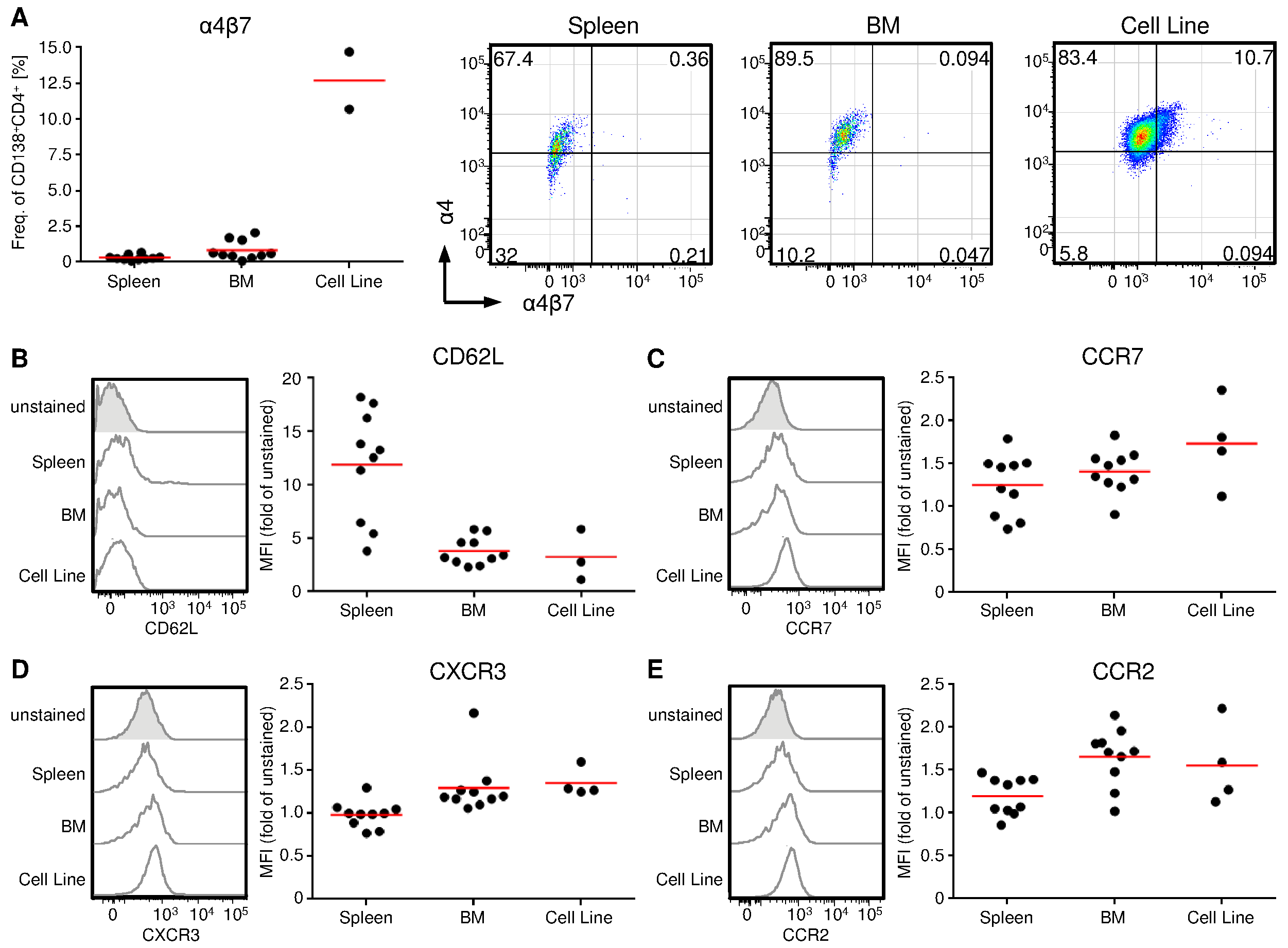

Supplement: Figure S2 — Flow cytometric measurement of surface receptors associated with BM homing and infiltration of myeloma cells. MOPC-315.BM luc+ myeloma cells were either directly taken from cell culture or extracted from BM and spleen as indicated and identified as CD138+CD4+ double positive cells. α4β7 integrin positive MOPC-315.BM luc+ cells were identified by flow cytometry as α4+ (CD49d+) and α4β7+ double positive. Representative quadrant gates or histograms for each organ and cell line, including unstained fluorescence minus one (FMO) sample are shown. Graphs state the frequency within CD138+CD4+ MOPC-315.BM luc+ cells expressing α4β7 (A) or fold difference of mean fluorescence intensity (MFI) values of CD62L (B), CCR7 (C), CXCR3 (D) or CCR2 (E) in relation to the unstained FMO sample. (TIF) [file pone.0052398.s002.tif]

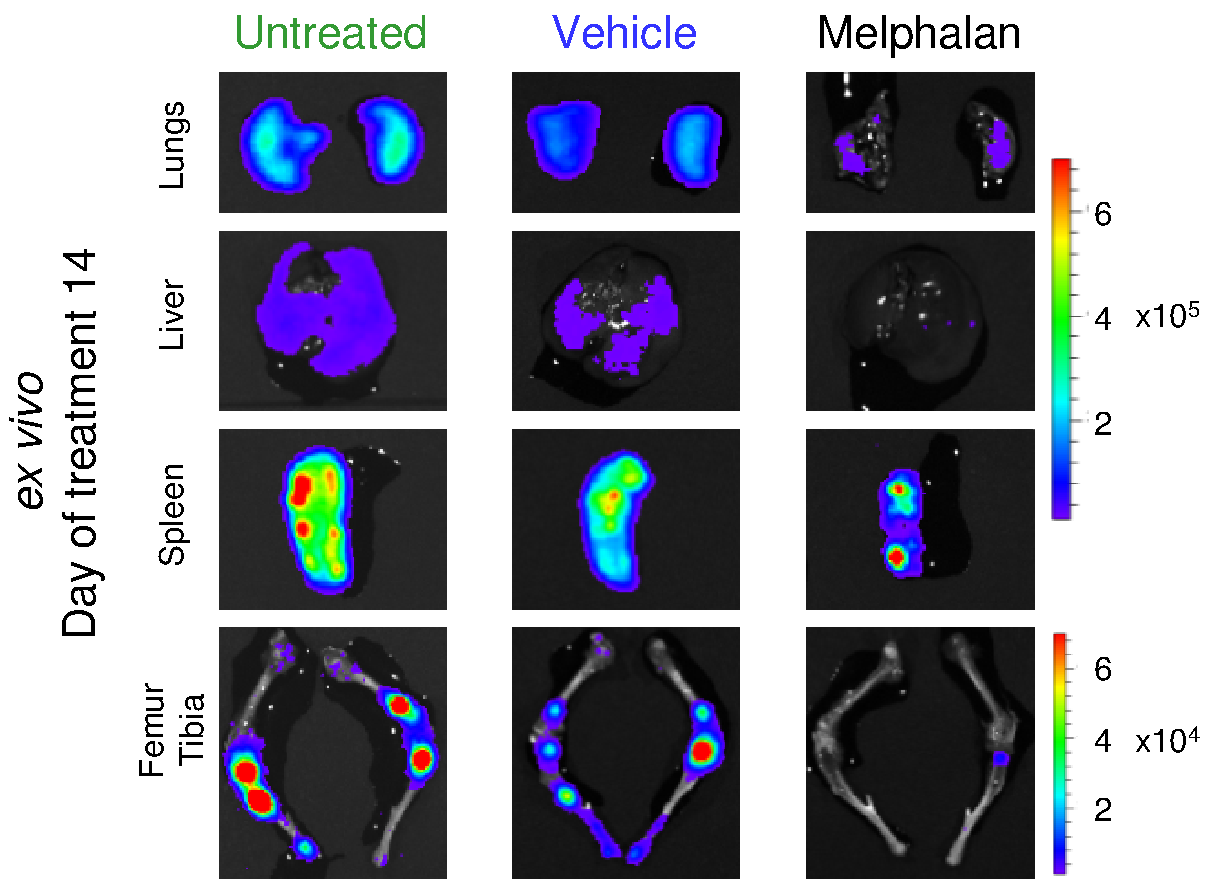

Supplement: Figure S3 — Verification of in vivo signal localization by ex vivo BLI. BALB/c wild type mice were injected with 1×10^5^ MOPC-315.BM luc+ cells via the tail vein. 19 days after inoculation tumors were established in all mice and readily detected by BLI. Then treatment was started ( = day 0 of treatment). Mice received 5 mg/kg melphalan or mock treatment (vehicle control) intraperitoneally. One control group of MOPC-315.BM luc+ tumor bearing mice did not receive any treatment (untreated). Mice were sacrificed on day +14 of treatment and organs were prepared for ex vivo BLI. Organs from one representative mouse per group are shown. In vivo BLI localization of signals from the liver, spleen and femur/tibia are confirmed. The signal from the lungs is only detected by ex vivo but not by in vivo BLI. Organs from the melphalan treatment group displayed lower signal intensities, indicating lower tumor burden when compared with untreated or vehicle controls. Therefore, ex vivo BLI corroborates in vivo data as well as histopathological analysis of the response to melphalan therapy. (TIF) [file pone.0052398.s003.tif]

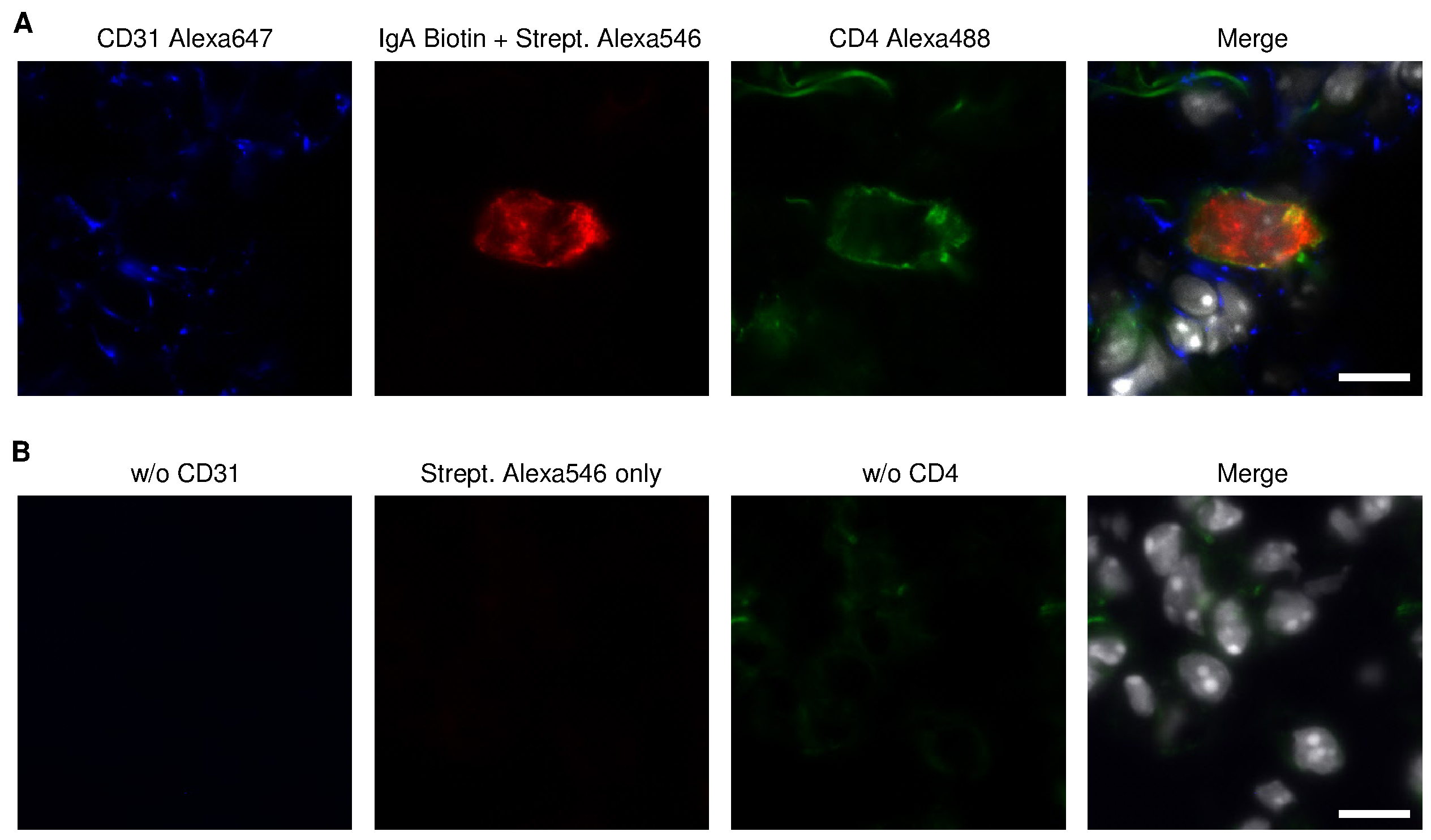

Supplement: Figure S4 — CD31 staining verifies MOPC-315 localization inside blood vessels in the lung. (A) Representative immunofluorescence staining of an IgA+CD4+ MOPC-315.BM cell within a pulmonary CD31+ vessel in the lung taken from untreated mice 33 days after MM injection. (B) Negative control staining, without anti-CD31, anti-IgA and anti-CD4. Only secondary strepavidin Alexa546 and DAPI was added. Single color channels and merge including DAPI are shown. Blue – CD31, red – IgA, green – CD4, white – DAPI (nuclei). Scale bar is 10 µm. Original magnification 400×/1.30 NA. (TIF) [file pone.0052398.s004.tif]

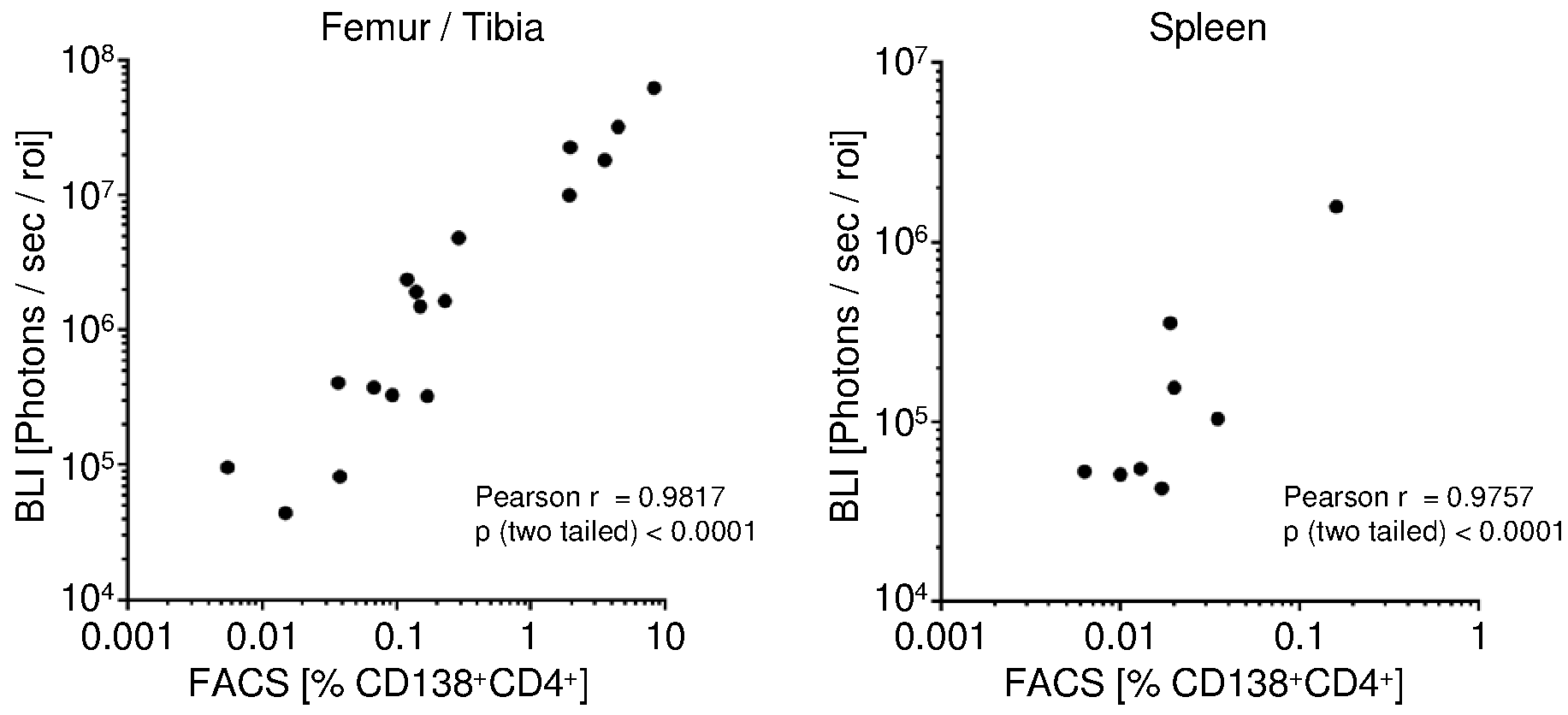

Supplement: Figure S5 — In vivo BLI measurements strongly correlate with MM burden determined by flow cytometry in spleen and femur/tibia. To additionally verify that non-invasive BLI data correlate with actual MM load we measured in vivo BLI signals from the spleen and femur/tibia and subsequently extracted cells from both organs for FACS analysis. For FACS MM cells were identified among living cells as CD4+CD138+. The measured percentage of MM cells infiltrating the spleen or bone marrow compartments was correlated to measured BLI signals using a Pearson correlation. Femur/tibia: Pearson r = 0.9817, p (two tailed) <0.0001; Spleen: Pearson r = 0.9757, p (two tailed) <0.0001. (TIF) [file pone.0052398.s005.tif]

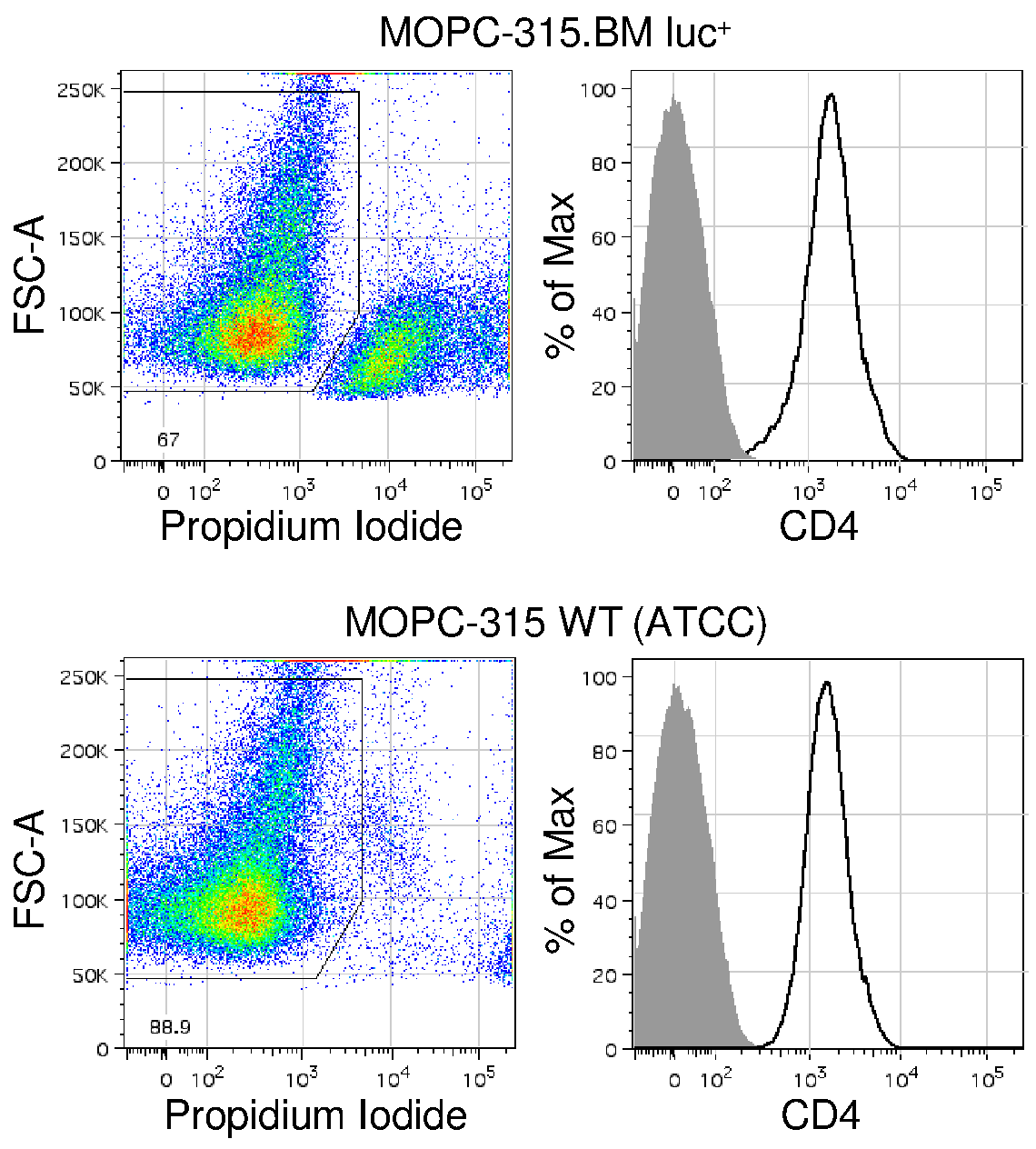

Supplement: Figure S6 — CD4 expression of MOPC-315.BM luc+ and the wild type (WT) cell line. MOPC-315.BM luc+ and MOPC-315 WT cells (obtained from ATCC) were stained for CD4. Only live cells as determined by propidium iodide staining were used for the analysis. Both cell lines clearly expressed CD4 to the same extent. Therefore, the constitutive CD138 and CD4 co-expression can be considered as a hallmark to uniquely identify these cells. Grey tinted histogram shows unstained luc+ or WT cells respectively, black histogram shows CD4 stained cells. (TIF) [file pone.0052398.s006.tif]
